# Mushrooms as Nutritional Powerhouses: A Review of Their Bioactive Compounds, Health Benefits, and Value-Added Products

**DOI:** 10.3390/foods14050741

**Published:** 2025-02-22

**Authors:** Akruti Singh, Ramesh Kumar Saini, Amit Kumar, Prince Chawla, Ravinder Kaushik

**Affiliations:** 1 School of Health Sciences and Technology, UPES, Dehradun 248007, Uttarakhand, India; akruti.singh0898@gmail.com (A.S.); rameshkumar.saini@ddn.upes.ac.in (R.K.S.); kumar.amit@ddn.upes.ac.in (A.K.); 2 Department of Food Technology and Nutrition, Lovely Professional University, Phagwara 144411, Punjab, India; princefoodtech@gmail.com

**Keywords:** functional foods, edible fungi, bioactive compounds, health benefits, value-added products

## Abstract

Mushrooms are known to be a nutritional powerhouse, offering diverse bioactive compounds that promote and enhance health. Mushrooms provide a distinguishable taste and aroma and are an essential source of vitamin D_2_, vitamin B complex, hydroxybenzoic acids (HBAs) and hydroxycinnamic acids (HCAs), terpenes, sterols, and β-glucans. Edible mushroom varieties such as *Hericium erinaceus*, *Ganoderma* sp., and *Lentinula edodes* are recognized as functional foods due to their remarkable potential for disease prevention and promotion of overall health and well-being. These varieties have antioxidants, anti-inflammatory, cytoprotective, cholesterol-lowering, antidiabetic, antimicrobial, and anticancer properties, as well as controlling blood pressure, being an immunity booster, and strengthening bone properties. In addition, they contain essential non-digestible oligosaccharides (NDOs) and ergothioneine, a potential substrate for gut microflora. Supplementing our daily meals with those can add value to our food, providing health benefits. Novel edible mushrooms are being investigated to explore their bioactive substances and their therapeutic properties, to benefit human health. The scientific community (mycologists) is currently studying the prospects for unlocking the full health advantages of mushrooms. This review aims to promote knowledge of mushroom culturing conditions, their nutritional potential, and the value-added products of 11 varieties.

## 1. Introduction

The Indian system of medicine, Ayurveda, emphasizes that “When diet is wrong, medicine is of no use. When diet is correct, medicine is of no need”. In this context, mushrooms can be vital in providing a balanced and wholesome diet [1]. Mushrooms, a kind of edible fungi that form sizable, firm, or robust fleshy structures, are found abundantly across the globe. Approximately 16,000 different types of edible mushrooms have been identified. Nearly 7000 varieties are recognized for their excellent taste and nutritional profile, and nearly 3000 are regularly included in daily food menus [2]. Basidiomycetes, particularly those found in the order Agaricales, are among the most notable in the diverse world of mushrooms. The structure of mushrooms includes several key components: mycelium, hypha, cap, lamellae, spores, stem, voula, and rings [3]. Mushrooms have an excellent nutritional profile and so, for centuries, mushrooms have been integrated into meals and their medicinal benefits for overall well-being leveraged [4]. It is estimated that there are nearly 1.5 million different species of fungi, among which scientists have identified around 110,000 types. Numerous mushrooms offer a wealth of flavor and essential nutrients; however, caution is paramount as some species are poisonous [5]. Mushrooms have historically been used in Traditional Chinese Medicine, for medicinal purposes for 3000 and 7000 years. For example, shiitake mushrooms, scientifically known as *L. edodes* (Berk.) Pegler, have been utilized both in nutrition and medicine since 600–1000 B.C. [6]. These fungi produce bioactive compounds such as peptides, sterols, polysaccharides, proteins, and phenols, which can be considered potential drugs [7]. Due to their rich nutritional profile and organoleptic properties, mushrooms are often blended into various dishes and can also serve as a meat substitute [8]. The nutritional profile is significantly influenced by the substrate a mushroom feeds on, environmental parameters, and its stage of maturity. Mushrooms contain various carbohydrates such as glycogen, xylose, mannose, galactose, glucose, and some insoluble forms like fiber, mannan, cellulose, and chitin. They also have a valuable compound known as glucan, which is characterized by glycosidic bonds at β (1, 3), β (1, 4), and β (1, 6), making mushrooms an excellent addition to a healthy diet [9]. With a unique umami flavor, mushrooms are consumed as part of everyday food dishes while enhancing their nutritional value [10]. As Kaul states, “Medicines and food have a common origin” [11].

This review aims to promote complete knowledge of mushroom culturing conditions and the nutritional potential of different varieties like *Agaricus bisporus*, *Calocybe indica*, *Volvariella volvacea*, *Auricularia polytricha*, *Schizophyllum commune*, *G. lucidium*, *Pleurotus ostreatus*, *Grifola frondosa*, *H. erinaceus*, *Flammulina velutipes,* and *L. edodes*. Their health benefits include anti-inflammatory, antioxidant, antidiabetic, antimicrobial, enhanced gut microbiota, and healing properties. In addition, we explore the mushroom value-added products available on the market. By consolidating information about the structures, bioactive compounds, and diverse uses of mushrooms, this article underscores the importance of mushrooms as a unique and valuable food source, which contributes to overall health and well-being.

## 2. Culturing Conditions

Mushroom fructification, the process of producing fruiting bodies, is initiated by a mature mycelia network. Many mushrooms are saprophytic fungi, acting as crucial decomposers in diverse ecosystems. For successful cultivation, the mushroom-growing conditions must be carefully optimized, and the substrate composition and formulation vary according to the species being cultivated [12]. Researchers are increasingly using agro-industrial wastes as substrates, including agricultural and industrial residues with low nitrogen contents [13,14]. Substrates also include organic materials such as cereal by-products (bran and shell), soybean meal, and compost, as well as inorganic supplements like ammonium salt and fertilizers, which provide the necessary nitrogen for mushroom cultivation [15,16]. Additionally, pulses, maize, soybean, sorghum, and residues from oil seeds, sugarcane, and cotton can be utilized as organic substrates. Even sawed wood residues are well-known substrates supporting mushroom growth and fruiting [10]. Some studies have utilized tea waste as a substrate for cultivating oyster mushroom varieties [17]. The roles of intrinsic and extrinsic factors are of equal significance in mushroom cultivation. Intrinsic factors include the carbon and nitrogen contents of the growth medium, pH level, and appropriate nutrition media, while extrinsic factors encompass temperature, humidity, light, and gas concentrations (CO_2_) [18,19,20]. The cultivation process enhances the economic use of agricultural residue to produce mushrooms and improves the relationships between fungal hyphae, substrates, and soil systems [21]. Several studies explored the use of synthetic or semisynthetic media as substrates for mushroom mycelium cultivation. Artificially synthesized media can supply the necessary nutrients for growth, including SDA (Sabouraud dextrose agar), MYE (malt yeast extract), YMEA (yeast malt extract agar), and PDA (potato dextrose agar). Additionally, enriched potato culture media like YPDA (yeast potato dextrose agar), PMA (potato malt agar), CMA (corn meal agar), PDYA (potato dextrose yeast agar), PM (potato malt peptone), PCA (potato carrot agar), PGA (potato glucose agar), and PSG (potato sucrose gelatin) have been noted in studies as effective substrates for mycelium culturing. The analysis showed that synthetic media such as PDA and MEA provide the maximum nutrient effect due to their rich nutrient composition, which is essential for optimal fungal growth. The influence of culture media on mycelia development varies based on the fungal species and strains. Nutrient media like PDA or PGA, followed by MEA and MCM, were identified as the best options for promoting mycelia development. However, agriculture and food wastes contain a significant number of natural-based chemicals, which can result in greater biomass growth than synthetic and semi-synthetic media. The optimal cultivation temperature for mycelia is often linked to the fungus’s genetic origin and the environmental conditions in which it naturally grows. Most basidiomycetes thrive at temperatures between 20 and 30 °C, while some species prefer higher temperatures ranging from 35 to 37 °C [14,19,22].

## 3. Nutritional Potential of Mushroom

Edible mushrooms are grown with a mini packet of essential nutrients, which include a good amount of water, carbohydrates, protein, lipids, fibers, macronutrients, and micronutrients [23,24]. Mushrooms contain both primary and secondary metabolites. Primary metabolites are responsible for energy production, while secondary metabolites are responsible for medicinal properties [1]. These macrofungi’s organoleptic features and high nutritional content contribute to their growing popularity. Edible mushrooms are rich in proteins and account for 19–35% of the dry mass, while carbohydrates constitute 50–65% of the dry mass, rendering mushrooms an abundant source of high-quality dietary fiber. Furthermore, mushrooms exhibit a low lipid content, ranging from 2% to 6% of the dry mass, and are regarded as hypocaloric [25]. These powerful compounds provide numerous health benefits including antimicrobial defense, protection against oxidative damage, and anti-inflammation properties. Moreover, mushrooms exhibit antidiabetic, anticancer, antiviral, and anti-immunomodulatory activities, making them valuable ingredients in the development of functional foods [26,27,28]. Mushrooms also contain many bioactive compounds including alkaloids, ergosterols, polysaccharides, polyphenols, terpenoids, lectins, glycoproteins, sesquiterpenes, sterols, and lactones. The concentrations of these bioactive chemicals vary considerably based on several parameters, including culture, strain, storage conditions, substrate, and processing conditions [29]. The nutritional compositions of some edible mushrooms are presented in Table 1.

### 3.1. Carbohydrates

Mushrooms are low-calorie foods owing to low carbohydrate contents, minimal sugar levels (no glucose), and high fiber contents. They contain various carbohydrates including simple sugars like sucrose, xylose, glycogen, rhamnose, mannose, fructose, galactose, mannose, and xylose, and polysaccharides such as cellulose, glycoproteins, α-glucans, and β-glucans, glucan, mannoglucan, heteroglycan, galactomannan, and lentinan [41,42]. Mushrooms are also high in dietary fibers, primarily non-starch polysaccharides, with 4 to 9% being soluble and 22 to 30% insoluble [43]. They contain non-digestible carbohydrates, such as chitin and (1→3)-β-d-glucans, which promote intestinal health, and the main components of the cell wall are β-glucans and polysaccharides. Additionally, mushrooms contain non-digestible oligosaccharides (NDOs) consisting of carbohydrate molecules of fewer than 20 monosaccharide units joined by glycosidic linkages. These NDOs are resistant to hydrolysis by salivary and intestinal digestion enzymes associated with various beneficial advantages, including antipathogenic and prebiotic characteristics. Individuals can increase their intake of NDO through food sources, like mushrooms, and supplements derived from dried fruiting bodies or mycelium-based products from fungal species [44,45,46]. Although mushrooms have less fiber than vegetables and fruits, they are still a nutritious, low-energy dietary option, particularly beneficial for type II diabetes and those seeking weight loss. Due to their low glycemic index (GI) and glycemic load (GL), they do not cause spikes in blood sugar levels [47,48]. In *L. edodes*, key polysaccharides such as emitanin, lentinan (a β-(1,3)-D-glucan enhances the effectiveness of chemotherapeutic drugs), and KS-2 are found to benefit health [49].

### 3.2. Protein

Edible mushrooms are often high in protein, although the protein content varies widely depending on the mushroom’s species, stage of growth, and growth medium. They contain essential amino acids such as lysine, valine, tryptophan, isoleucine, methionine, leucine, and threonine. Mushrooms also provide proteins such as lectins, laccases, histidine, phenylalanine, and cysteine [50,51,52,53]. Notable mushrooms of the *Pleurotus* species have high-quality protein due to the effective distribution of essential and non-essential amino acids such as gamma-aminobutyric acid (GABA), a critical neurotransmitter [50]. When compared to other food sources, the protein content of edible mushrooms is quite competitive [54]. Animal-based foods (dry weight) contain protein, which is present in proportions of at least 27% for milk, 37–83% for meat, 53% for eggs, and the highest 58–90% for fish and crustaceans [55]. In contrast, plant-based sources such as legumes contain 22–40%, cereals 8–18%, nuts 4–20%, other seeds 18–32%, and tubers less than 10% [56,57]. Certain species of edible fungi, *A. bisporus* 32.10%, *H. erinaceus* 22.30%, and *L. edodes* 22.80%, provide protein concentrations that are equal to or exceed those found in animal-derived sources such as dairy products, meat, eggs, and seafood [54,58,59,60,61]. Consequently, these edible fungi represent an exceptional source of high-quality protein that is more accessible, cost-effective, and exhibits a reduced environmental footprint, and so, in the future, they will become a compelling alternative to both animal-derived and various plant-based protein options [62]. Ergothioneine (EGT) is uniquely sulfur-containing and has excellent free radical scavenging activity. EGT is found in high concentrations in mushroom species like hen of the woods, shiitake, King Boletes, Enokitake, and oyster mushrooms [62,63,64,65]. It has been correlated with several health benefits, including lower rates of dementia and cardiovascular disease and anti-inflammatory and cytoprotective effects, and it may even lead to a longer life expectancy. Mushrooms have much greater quantities of ergothioneine than cereals, vegetables, and meat [64,66]. As such, edible mushrooms are appealing foods with significant nutritional benefits that contribute to overall health.

### 3.3. Fats

Edible mushrooms represent a low-calorie aliment with a minimal fat content (4–6%). *A. bisporus,* also known as the button mushroom, has a total fat content ranging from 0.34 to 2.2 g per 100 g of dry weight [67,68]. The three major fatty acids present in edible mushrooms are linoleic acid (C18:2), oleic acid (C18:1), and palmitic acid (C16:0). Linoleic acid is useful in reducing the amount of lipids in the blood and helping to alleviate arthritis symptoms [50]. Additionally, these fungi are abundant in polyunsaturated fatty acids (PUFAs), particularly oleic (1.1–12.3 g/100 g fresh weight (FW)), stearic (1.6–3.1 g/100 g FW), palmitic (10.3–11.9 g/100 g FW), and linoleic acids. Ergosterol (ergosta-5,7,22-trien-3b-ol) is identified as the most prevalent sterol within edible mushrooms [68]. Although mushrooms are characterized by low caloric and fat contents, they exhibit a markedly elevated ratio of polyunsaturated fatty acids in comparison to saturated fatty acids [69].

### 3.4. Micronutrients

Mushrooms are rich in various vitamins such as vitamin B complex (B_1_, B_2_, B_3_, B_9_, and B_12_), vitamin C, vitamin D_2_, and vitamin E and minerals like calcium, cadmium, magnesium, phosphorus, iron, sodium, cobalt, zinc, potassium, copper, titanium, selenium, and molybdenum [50,70,71]. Tocopherol (α, β, γ, and δ) is a vitamin present in various mushroom varieties [24,71,72]. Mushrooms are known for their high potassium content and low sodium content, as potassium reduces tension in blood vessels and eventually helps in lowering blood pressure [70]. *A. bisporus* is particularly high in Na, Li, and K, but poor in Cu, Mn, Cr, Co, Pb, Ni, and Zn [73]. *H. erinaceus* contain high levels of K, P, and Mg followed by Na, Fe, Ca, Zn, Al, Cu, Li, Mn, and Ba. *G. lucidium* contains high levels of K, P, and Ca, followed by Mg, Na, Fe, Al, B, Zn, and Cu, and the least Mn [74].

### 3.5. Bioactive Compounds

**Figure 1:**
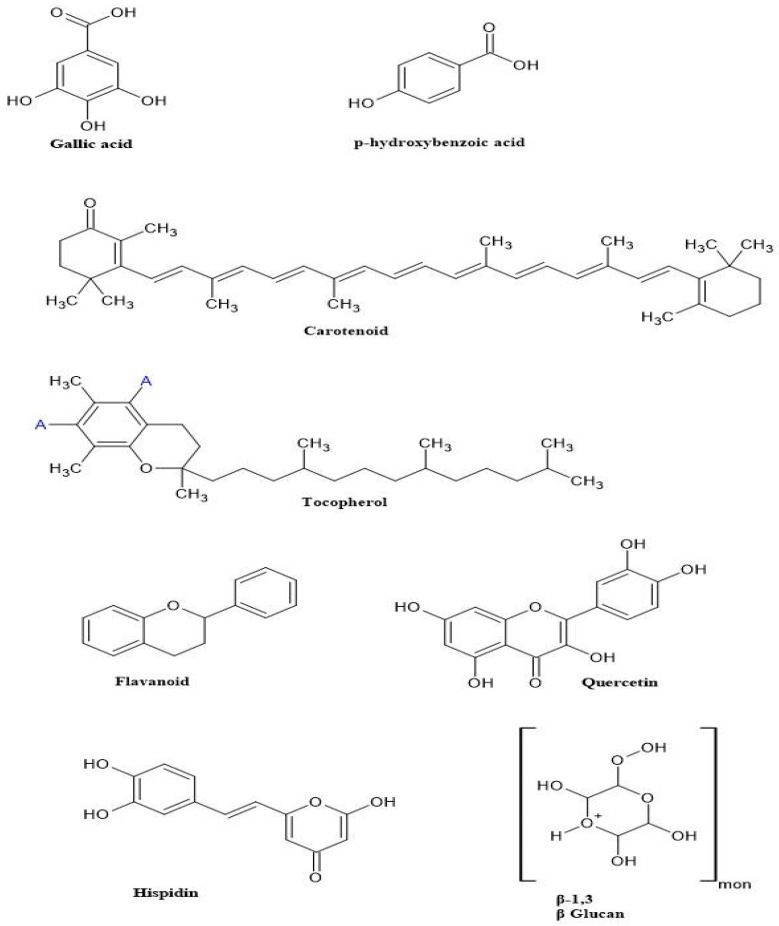
Structures of active compounds in the mushroom [71,81].

Mushrooms encompass a diverse array of bioactive constituents, which include phenolic acids, glycosides, volatile substances, alkaloids, flavonoids, organic acids, and a variety of biological catalysts such as amylases, cellulases, laccases, lipases, pectinases, proteases, phytases, and xylanases. The phenolic constituents identified within mushrooms comprise gallic acid, p-coumaric acid, caffeic acid, p-hydroxybenzoic acid, protocatechuic acid, and pyrogallol [75,76]. The majority of phenolic acids in mushrooms are hydroxybenzoic acids (HBAs) and hydroxycinnamic acids (HCAs). HBAs may be found in complex compounds such as tannins, lignin, and organic acids, whereas HCAs are attached to cell wall components such as lignin, cellulose, and protein. The most prevalent HCAs encountered in mushrooms include ferulic, sinapic, caffeic, and p-/o-coumaric acids, which play critical roles in lignin biosynthesis, disease resistance, and growth regulation [77,78]. Mild alkaline hydrolysis is the most effective for extracting them. In mushrooms, mainly quinic acid esters, and also gallic, gentisic, homogentisic, p-hydroxybenzoic, protocatechuic, 5-sulphosalicylic, syringic, vanillic, and veratric, are the most often observed HBA derivatives [24,71,72]. Anthocyanidins, biochanin, flavanols, flavones, isoflavones, flavanones, catechin, chrysin, myricetin, hesperetin, naringenin, naringin, formometin, resveratrol, quercetin, pyrogallol, rutin, and kaempferol are among the flavonoids present in mushrooms [72]. The structures of active compounds in mushrooms are presented in Figure 1. The quantity of these bioactive chemical compounds in mushrooms depends on the substrate, culturing conditions, storage conditions, and cooking procedures [48]. *G. lucidum* produces several terpene derivatives, including ganoderal, ganoderic acids, lucidone, ganodermanondiol, ganodermic, and ganodermanontriol [49]. *H. erinaceus* is known for hericenones (A-J, I, L, and K), erinacines (A-K, P-V, Z1, and Z2) [79,80], and *L. edodes* contains a polysaccharide known as lentinan [81]. The bioactive molecules, health benefits, and food products prepared from mushroom varieties are presented in Table 2.

## 4. Therapeutic Efficacy of Mushrooms

Medicinal mushrooms are rich in bioactive compounds such as phenolic acids, lectins, β-glucans, polysaccharides, and terpenoids, offering several health benefits that can significantly enhance the quality of life [107,108]. These compounds possess a wide range of properties including prebiotic, immune-modulating, antioxidant, hepatoprotective, anti-inflammatory, antihyperlipidemic, cytotoxic, anticancer, antioxidant, hypocholesterolemic, antidiabetic, antiallergic, antiviral, antibacterial, antiparasitic, antimicrobial, antifungal, radical scavenging, cardiovascular, wound healing, and detoxification effects [27,60,106,109,110,111]. Numerous mushroom varieties are recognized for their medicinal properties. For example, *G. lucidum* is often referred to as the ‘king of medicinal mushrooms’, along with *L. edodes* (shiitake) and *G. frondosa* (maitake), which are widely used for medicinal purposes across many regions of Asia [77,105,112]. In vitro research, in vivo experimentation, and clinical trials involving human subjects have elucidated that mushroom extracts and fresh consumable fungi provide an extensive range of therapeutic advantages, as elaborated upon in the subsequent discussion.

### 4.1. Anti-Inflammation Property

Inflammation is a defense mechanism in which the blood flow increases to the site of tissue infection, playing a crucial role in the healing process by eliminating harmful cells [113]. However, inflammation also leads to the destruction of cells, which is necessary for recovery. Mushrooms possess properties that allow them to act directly on inflammation. Their lipids, rich in unsaturated fatty acids, exhibit anti-inflammatory qualities as these fatty acids are precursors of eicosanoids involved in balancing inflammatory and anti-inflammatory processes [114]. Mushroom taxa such as *Agaricus* sp., *Pleurotus* sp., and *Termitomyces* sp. exhibit a high abundance of polysaccharides and synthesize biomolecules that play a pivotal role in the protection of joints against inflammatory mechanisms [83,115]. A study was conducted on the mushroom variety *Cordyceps* spp. containing the nucleoside compound cordycepin, which stimulates the generation of interleukin 10; as a result, it is an anti-inflammatory cytokine compound [116]. *H. erinaceus* has also been shown to have anti-inflammatory effects that were demonstrated for both *H. erinaceus* and *H. echinacea*-derived erinacine A, which protect against brain-ischemia-induced neuronal cell death in rats. The mechanism was the suppression of iNOS and MAPK, lowered proinflammatory cytokines, and the mushroom’s nerve development capabilities [117].

### 4.2. Healing Property

Healing is categorized into four stages: hemostasis involving blood clotting, inflammation, proliferation pertaining to tissue growth, and maturation encompassing tissue re-modeling. The repair process is complex and involves various cellular mechanisms such as epithelial cell stimulation, cytokine release, and growth factors. The extract and metabolites from varieties like *G. lucidum* and *A. blazei* (polysaccharides) showed wound-treating properties, including different mechanisms such as epithelial cell stimulation, cytokines, and growth factor release [118]. Chitinous polymers were extracted from the common *A. bisporus* mushrooms by employing straightforward methodologies and subsequently transformed into continuous fibers utilizing a specially designed laboratory-scale fiber-spinning apparatus. The resultant spun fibers consist of an array of chitin fibrils embedded within a glucan matrix, with their fiber dimensions meticulously governed by the specifications associated with needle gauges. After 30 s of contact with a small amount of water (<10 μL), all mushroom chitin fibers demonstrated self-healing characteristics. A microblade may successfully restore macroscopically injured mushroom chitin strands’ natural form and tensile characteristics, as indicated by the enhanced self-healing capability for tensile strength (reaching 119%) and breaking strain (attaining 132%). This implies that the process of swelling and deswelling of mushroom chitin fibers may have resulted in the interlocking of chitin fibrils and glucan across the impaired fiber surfaces, resulting in significant self-healing activity [118]. A study was conducted on *G. luciderma* in rats, in which indomethacin caused stomach mucosal lesions, and the polysaccharide fraction induced peptic ulcers for healing in rats [27].

### 4.3. Enhancing Gut Microflora

**Figure 2:**
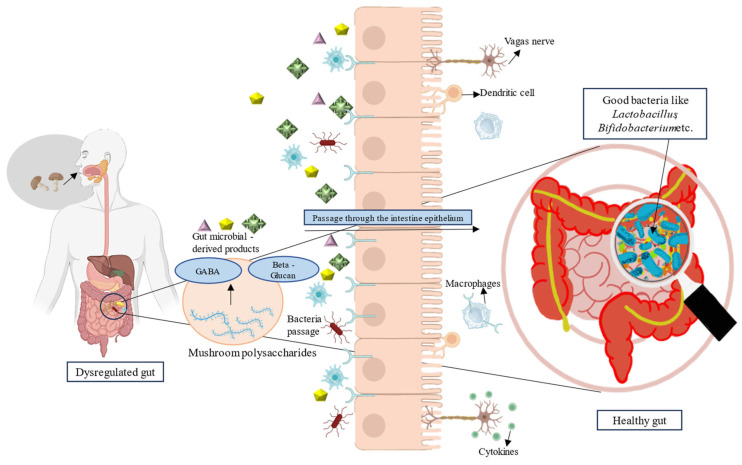
Mushrooms as a potential prebiotic.

Prebiotics are “a substrate that is selectively utilized by host microorganisms conferring a health benefit” [119]. Mushrooms are valuable sources of prebiotics including polyphenols, oligosaccharides, and fibers, which enhance the metabolic activity of beneficial members of gut microflora [117]. A mushroom *G*. *lucidum* contains polysaccharides and peptides that are non-digestible by pathogens, preventing their multiplication and thereby altering the gut microbiota [11,113]. These indigestible polysaccharides derived from mushrooms serve a prebiotic role, suppressing the proliferation of pathogenic bacteria within the gastrointestinal tract while enhancing the growth of beneficial probiotic bacteria. *G. lucidum*, *H. erinaceus*, *L. edodes*, and *G. frondose* are among the most frequently reported edible mushrooms known to modulate gut flora [119,120]. β-glucan, a type of polysaccharide found in mushrooms, can be fermented by gut bacteria, leading to beneficial changes in the host’s microbiome [49]. The diagrammatic representation of how a mushroom-based diet enhances gut microflora is depicted in Figure 2.

### 4.4. Anticancer Properties

**Figure 3:**
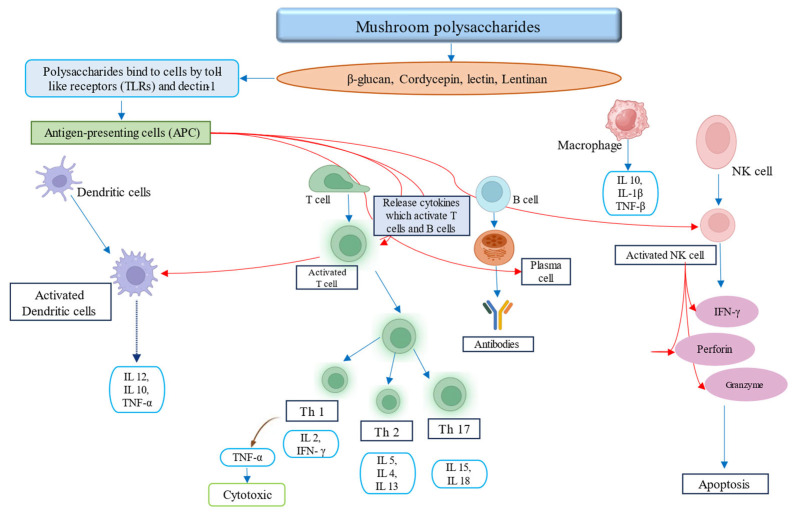
Mushrooms contain certain polysaccharides for the immune response.

Cancer is a fatal disease causing over 10 million deaths yearly according to the World Health Organization (WHO). Research has demonstrated that polysaccharides derived from mushrooms can inhibit tumor progression by enhancing the immune response, particularly through their impact on natural killer (NK) cells and macrophages via T-cell activation and cytokine secretion [121]. Polysaccharides from mushrooms can impede tumor progression by augmenting the immune response through their influence on natural killer cells and macrophages mediated by T-cell activation and cytokine secretion [122,123,124]. Notably, nearly 200 species of edible mushrooms demonstrated the capacity to reduce the growth of various cancer cells [125]. Specific compounds found in different mushroom species have been identified for their antitumor properties. For instance, *A. bisporus* contains quinone 490 and 1-oleoyl-2-linoleoyl-3-palmitoyl glycerol, and ganoderiol F and ganodermanontriol in *G. lucidum* and galactoxyloglucan in *H. erinaceous* have shown potential in combating cancer [68]. Mushrooms are rich in various anticancer components such as antroquinonol, krestin, cordycepin, lectin, sulfated polysaccharide hispolon, lentinan, and maitake D fraction [121,126]. The polysaccharide β-glucan is acknowledged for its role in augmenting immune functionality through the stimulation of cytokine synthesis, which subsequently triggers the activation of both phagocytes and leukocytes [80,127]. *L. edodes* contains lentinan and lectins, which demonstrated cytotoxic effects on breast cancer cells [128]. Studies indicate that hispolon, an active polyphenol compound, demonstrates potent antineoplastic effects through multiple mechanisms, including the upregulation of death receptors and downregulation of antiapoptotic proteins like c-FLIP, Bcl-2, and Bcl-xL. Furthermore, hispolon enhances the effectiveness of chemotherapeutic agents, making it a promising candidate for cancer therapy [129]. Furthermore, *G. lucidum* contains certain polysaccharides that are beneficial for mitigating colorectal cancer symptoms as they reduce the expression of rectal cancer-related genes. These polysaccharides also demonstrate cancer-preventive and therapeutic actions by dynamically controlling the gut microbiota and host immune responses. *G. lucidum* polysaccharides can modulate the immune system by activating and expressing cytokines related to inflammation (e.g., interleukin-1, interleukin-6, and tumor necrosis factor-α) and antitumor activity (e.g., interferon-γ and tumor necrosis factor-α). A contemporary in vivo study underscored that a newly identified acid-soluble polysaccharide extracted from *G. frondosa* exhibited protective effects on the thymic and splenic tissues of mice with tumors, concurrently inhibiting the proliferation of H22 solid tumors. These bioactive compounds markedly enhanced the functional activities of natural killer (NK) cells, macrophages, CD19+ B cells, and CD4+ T cells, ultimately facilitating apoptosis of H22 cells through the induction of G0/G1-phase cell cycle arrest [123]. A diagrammatic representation of the mushroom polysaccharides working as anticancer agents is presented in Figure 3.

### 4.5. Antioxidant Properties

**Figure 4:**
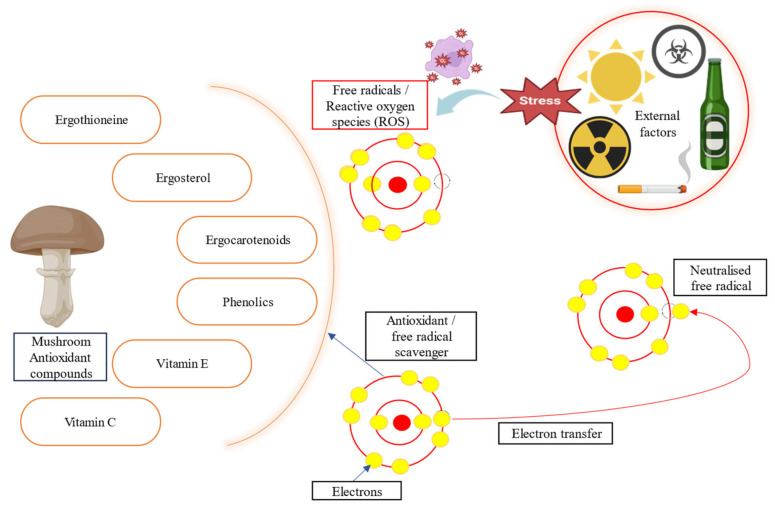
The antioxidant power of mushroom.

Oxidative stress can damage DNA, protein, and cell membranes, which eventually leads to various major diseases such as tumors, diabetes, neurodegenerative diseases, and kidney disease [129]. Polysaccharopeptides found in mushrooms can improve overall fitness by triggering enzymes that remove free radicals and reduce oxidative stress [87]. Mushrooms contain a variety of antioxidant compounds including ergothioneine, ergosterol, carotenoids, phenolics, tocopherols (vitamin E), ascorbic acid (vitamin C), polysaccharides (acidic polysaccharides), and amino acids [hydrophobic amino acids (HAAs) like leucine, isoleucine, valine methionine, proline, alanine, etc.] [123,130,131]. For example, *P. ostreatus* extract has been demonstrated to increase catalase gene expression and diminish free radical-induced protein oxidation in adult rats, protecting against age-related illnesses. The ethanolic extract of dietary *P. ostreatus* mushrooms inhibits lipid peroxidation, chelates ferrous ions, reduces ferric ions, and quenches 2,3-diazabicyclo. Another study attributed the superior antioxidant properties of *P. ostreatus* to its carbohydrate component—specifically, β-glucan—which may be responsible for its efficacy [24,132,133]. Furthermore, *P. ostreatus* mushrooms provide a wealth of antioxidants in food sectors, particularly as food additives [134]. An antioxidant assay determined the free radical scavenging activity of *A. bisporus* polysaccharide extracts. At 250 μg/mL, the extract displayed an 86.1% free radical scavenging activity, which was substantially greater (*p* < 0.01) than BHT (83%) [29]. As a result, mushroom consumption may enhance an individual’s antioxidative capacity, thereby reducing oxidative stress in the body [119]. The stabilizing of free radicals is shown in Figure 4.

### 4.6. Antidiabetic Properties

Antidiabetic compounds in various mushroom species typically exhibit the following effects: (1) prevention of β cells’ apoptosis and promotion of their regeneration; (2) regulation of glucose metabolism; (3) inhibition of inflammation and oxidation; and (4) enhancement of gut microbiota [109,135]. A study on the polysaccharide compounds of *G*. *lucidum* demonstrated that these compounds reduce insulin resistance without damaging pancreatic islet cells and successfully reverse the process of diabetes [136]. Mushroom extracts from *A. bisporus*, *G. frondosa*, *H. erinaceus*, *G. lucidum*, and *Pleurotus* species reduce blood glucose levels in the liver and muscle by controlling the expression of glycogen synthase kinase (GSK-3β), glycogen synthase (GS), and glucose transporter 4 (GLUT4). As a result, GSK-3β may be identified as a negative regulator that is modulated by insulin-mediated, GS-regulated activity [137]. A study was conducted on high doses of *A. bisporus* extract, which was orally administrated to decrease the severity of streptozotocin-induced hyperglycemia in Sprague–Dawley rats. The rats were provided *A. bisporus* powder (200 mg/kg of body weight) for three weeks, which resulted in a significantly decreased plasma glucose concentration (24.7%), triglyceride content (39.1%), alanine aminotransferase (11.7%), and aspartate aminotransferase (15.7%). Additionally, *G. frondosa* has been noted for its role in blood glucose regulation [138,139].

### 4.7. Antimicrobial Property

The mushroom species *P. ostreatus* is considered a medicinal mushroom due to its antimicrobial properties because of β-D glucan’s presence. It includes several antibacterial agents, such as phenolic compounds, phenolic acids, and flavonoids, which are beneficial in this variety and others [9]. Ethanol extracts from two grown mushroom kinds, *L. edodes* and *A. bisporus,* were tested for antibacterial activity against *Klebsiella pneumoniae*, *Staphylococcus aureus*, *Enterococcus faecalis*, and *Acinetobacter baumannii*. Upon exposure to extracts derived from *L. edodes* and *A. bisporus*, bacterial cell death was observed, attributable to the elevation of protein and DNA levels within the surrounding milieu, indicative of bacterial cell deformation in response to the extracts above. Developing various extracts to combat antibiotic-resistant bacteria is crucial as resistance is anticipated to become one of the most serious health issues in the future. Moreover, there is a significant gap in the literature discussion on the antimicrobial mechanism of mushroom-based compounds [140]. Studies on *P. ostreatus* have demonstrated its effectiveness against Gram-positive bacteria (*Bacillus cereus*, *Bacillus pumilis*, *Micrococcus luteus*, *E. faecalis*, *S. aureus*, and *Bacillus subtilis)* and Gram-negative bacteria (*Klebsiella oxytoca*, *K. pneumonia*, *Shigella* sp., *Salmonella pullorum*, *Salmonella typhi*, *Moraxella* sp., *Escherichia coli*, *Burkholderia pseudomallei*, *Vibrio* sp., and *Pseudomonas aeruginosa*). Moreover, it showed antibacterial action against *Fusarium oxysporum*, *Myrothecium arachidicola*, and *Penicillium rapiricol* [139]. Additionally, *A. bisporus* has demonstrated antibacterial properties against *Neurospora sitophila*, and *Lenzites betulina* has shown antibacterial action against *S. aureus*, *E. coli*, *B. subtilis*, *Fusarium graminearum*, *Gibberella zeae*, and *Cercosporella albo maculans. Trichoderma giganteum* has antibacterial action against *F. oxysporum*, *Myrothecium arachidicola*, and *Penicillium rapiricola. H. erinaceus* has antibacterial properties against *Helicobacter pylori* [141].

## 5. Value-Added Products

**Figure 5:**
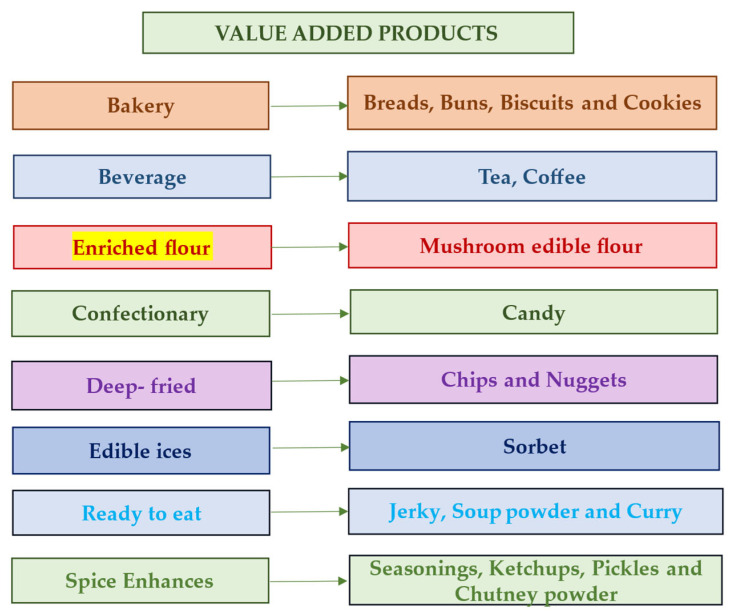
Value-added products prepared from edible mushrooms [105].

Several value-added products have been formulated from mushrooms, such as mushroom chips, mushroom soup powder, mushroom pickles, papad, cookies, bhujia, noodles, murabba, yogurt, dried mushrooms, canned mushrooms, mushroom pasta, mushroom kheer, fries, preserve, candies, and mushroom pakora. Additionally, medicinal products such as mushroom pills, mushroom tea, mushroom immunity booster, and protein powder are designed to satisfy taste preferences while providing essential nutrients and bioactive compounds [142,143]. Certain value-added products made from mushrooms are listed in Figure 5.

Value-added products such as muffins prepared with mushroom powder and white flour have been shown to increase protein, ash, crude fiber, and fat, making them healthier and more nutritious compared to traditional white flour muffins [140]. Functional mushroom cookies and biscuits were also formulated, containing higher nutritional values than those prepared from normal flour. Cookies prepared with *Cordycepes militaris* at concentrations of 1, 3, and 5%, respectively, exhibited increased phenolic and antioxidant contents. Additionally, these cookies showed higher levels of crude fiber, ash content, protein, and crude fat [142]. The incorporation of *C. militar* is flour caused the cookies to become softer, with the hardness slightly decreasing as the concentration of *C. militaris* flour increased (*p* > 0.05). The addition of *C. militaris* flour led to a distorted gluten network, which accounts for the decrease in hardness. Biscuits prepared with a combination of mushroom flour and wheat flour were shown to be more nutritious, with research showing that they can help control diabetes and treat protein–energy shortages. These biscuits also have low GI and GL. Similar to other value-added products, they showed an increase in protein content, ash content, crude fiber, and fat content [143]. The increased properties may be due to bioactive compounds found in wheat and mushroom flours that block alpha-amylase and alpha-glucosidase enzymes. Mushrooms’ high fiber content may help with hyperglycemia management. This conclusion is consistent with Owheruo’s (2023) discovery that a high-fiber diet lowers blood sugar levels. Nwosu (2022) discovered that those with type 2 diabetes who took a fiber-rich supplement had lower fasting blood glucose levels. According to these findings, mushroom biscuits have the potential to lower blood glucose levels, which may be advantageous for diabetic individuals and those managing hyperglycemia as well as other degenerative illnesses [142,143].

Bars with incorporated dried shiitake mushroom demonstrated hypocholesterolemia and hypoglycemic effects with no toxicity. When assessed for shelf life over 6 months, the bars indicated no significant changes in the microbiological parameters comprising coliforms, *S. aureus*, *B. cereus*, and *Salmonella* sp., with each sample containing fewer than 10 colonies of microorganisms [144]. Similarly, “papad”, a trendy dehydrated snack in the Indian market, was fortified with mushroom powder, which increased its protein content, dietary fibers, phosphorous, and calcium [136].

## 6. Conclusions

Both underdeveloped and developing countries are facing grave issues of malnutrition, poverty, and food insecurity. The consumption and production of highly functional foods, such as mushrooms rich in nutrients and bioactive compounds and offering protection against various diseases, is a step toward address these food issues, as they offer protective and therapeutic benefits against many diseases. The bioactive compounds in mushrooms make them highly suitable for consumption through different sources like food, nutraceuticals, and medicine. Adding mushrooms to our daily diet boosts our nutrient intake by providing essential macro- and micronutrients, and bioactive compounds that are lacking in regular meals. Additionally, this study emphasizes the significant role of mushroom polysaccharides, polyphenols, terpenoids, and glycoproteins in promoting gut health, supporting the immune system, and exhibiting anticancer, antidiabetic, and inflammatory activities. However, further research is needed to elucidate the precise mechanism underlying these health benefits in humans. An in-depth evaluation of mushroom products and varieties in various geographical regions should be performed and technological advancements made for their correct utilization as foods and bioactive agents. Moreover, the production of mushroom-based snacks, beverages, soups, and sauces remains limited on a large scale, and these products often have a short shelf life; therefore, further research is essential to fully understand the potential and limitations of mushroom-based products on the market.

## Figures and Tables

**Table 1:** Nutritional profiles of different mushroom varieties.

Mushroom Varieties	Content/100 g					Reference
	Carbohydrate	Protein	Fat	Fiber	Ash	
*Agaricus bisporus*	47.20	32.10	3.10	8.90	8.70	[30]
*Auricularia polytricha*	51.65	18.7	1.60	22.80	3.77	[31,32]
*Volvariella volvacea*	43.16	19.40	2.49	15.10	11.71	[33]
*Calocybe indica*	6.80	3.22	1.05	1.11	2.30	[34]
*Schizophyllum commune*	42.0	15.55	9.00	30.00	3.50	[35]
*Ganoderma lucidium*	37.33	8.54	1.91	50.19	2.03	[36]
*Pleurotus ostreatus*	61.10	20.00	2.50	7.90	7.70	[30]
*Flammulina velutipes*	42.83	20.26	4.50	23.31	9.00	[37]
*Grifola frondosa*	70.70	19.30	3.80	-	6.10	[38]
*Lentinula edodes*	64.40	22.80	2.10	1.26–2.95	6.00	[39]
*Hericium erinaceus*	57.00	22.30	3.50	3.30–7.80	-	[40]

**Table 2:** Bioactive molecules, health benefits, and food products prepared from mushroom varieties.

Mushroom Name	Common Name	Compounds	Health Benefits	Products	References
*Agaricus bisporus*	Button mushroom	Dietary fiber (chitin), sterols, amino acids, unsaturated fatty acids (linoleic and linolenic acids), phenolic acid (Ferulic acid, gallic acid, cinnamic acid, myricetin, caffeic acid, catechins, protocatechuic acid, p-coumaric acid, chlorogenic, sinapic, *p*-hydroxybenzoic, vanillic, salicylic, and syringic acids), and vitamins (vitamin B complex—vitamin B_1_, vitamin B_2_, vitamin B_3_, vitamin B_6_, vitamin B_7_, and vitamin B_12_; vitamin C; vitamin D2; vitamin E (γ-tocopherol, α-tocopherol, δ-tocopherol)), minerals (copper (Cu), cobalt (Co), iron (Fe), selenium (Se), potassium (K), manganese (Mn), phosphorus (P), calcium (Ca), zinc (Zn), iron (Fe), magnesium (Mg), and sodium (Na)), and indols (indole acetic acid, tryptamine, kynurenic acid, melatonin, serotonin and L–tryptophan)	Immunomodulatory and hepatoprotective activities are antimicrobial, antioxidant, antibacterial, antidiabetic, antitumor, anti-inflammatory, antihypertensive, anticancer, and antihypercholesterolemic	Medicinal, cosmetic purposes (lotions, creams, shampoos), functional bread, biscuits	[68,82,83,84,85,86]
*Auricularia polytricha*	Wood ear/Jew’s ear	Phytochemicals, glucan, rhamnose, fucose, arabinose, xylose, mannose, galactose, glucose, and antioxidants	Antioxidant, antitumor, antimicrobial, anti-inflammatory, and immunity boosting	Noodles, steamed white bread	[87,88,89]
*Volvariella volvacea*	Straw mushroom/paddy straw mushroom/Chinese mushroom	Beta-glucan, amino acids (leucine, isoleucine, lysine, methionine, tryptophane, valine, threonine, histidine, and phenylalanine), saturated fatty acids and unsaturated fatty acids, vitamins (vitamin C, vitamin B_1_, vitamin B_2_, vitamin B_3_, and vitamin B_7_), phenolic acids, tannins and flavonoids, terpenes, polypeptides, and steroids	Anti-inflammatory, hyperlipidemia treatment, anticancer, antioxidant, prevention of chronic hepatitis, antimalarial, antiallergic, and treatment of arteriosclerosis, cardiovascular diseases, and neurodegenerative diseases	Brown rice extruded snacks	[69,90]
*Calocybe indica*	Paddy straw milky mushroom	Includes 15 amino acids (most abundant glutamic acid and isoleucine), polyphenols (flavonoids, alkaloids, and triterpenoids), phenolics (gallic, vanillin, protocatechuic acid, naringin, naringenin, homogentisic acid, hesperetin, ferulic acid, caffeic acid, and formononetin), minerals (phosphorous (P), magnesium (Mg), barium (Ba), iron (Fe), aluminum (Al), manganese (Mn), copper (Cu), zinc (Zn), boron (Bo), nickel (Ni), chromium (Cr)), 17 fatty acids, vitamins (vitamin B, vitamin D, vitamin C, vitamin E), and β-glucans	Antiaging, physical injury treatment, infection control, cardiovascular disease treatment, antioxidant activity, antilipid peroxidation characteristics, antimicrobial, anticancer, antidiabetic, and immunomodulatory	-	[40,71,91,92]
*Schizophyllum commune*	Split gill pushroom	Polysaccharides (uronic acid) and monosaccharides (glucose, fucose, ribose, rhamnose, galacturonic acid, galactose, xylose, arabinose, and glucuronic acid)	Anti-inflammatory, immunomodulator, anticancer, and antioxidant	-	[93,94]
*Ganoderma lucidium*	Lingzhi or Reishi	Organic germanium, heteroglycans, amino acids, glycans, proteoglycans, steroids, polysaccharides, triterpenoids, ganodermanondiol, nucleotides, minerals, vitamins, adenosine beta-d-glucan, ganoderic acids, polyphenols, triterpenoids ergosterol, peroxide ganopoly, ganoderol, hetero galactan-protein complex, lucidimins A-D, lucidimine E, MD-fraction Protein of LZ-8, and phenols (kaempferol, hesperetin, trans-cinnamic acid, gallic acid, quercetin, and naringenin)	Antioxidant, anti-inflammatory, antiallergic, immunomodulating, antitumor properties, diabetes treatment, hepatitis treatment, hypoglycemic, sedative, antiviral (HIV-1), antidepressant, antihepatotoxic, antiosteoporosis, cholesterol biosynthesis inhibitor, dizziness and insomnia treatment, antiobesity, hepatoprotective activity, antidyslipidemia, cardioprotective, neuroprotective, antiepileptic, nootropic, anxiolytic, and radioprotective	Enriched yoghurt, fat replacer to improve cakes, instant Reishi herbal mushroom tea, moon juice spirit dust, *Ganoderma* cell-repairing antiaging face mask, night cream	[11,40,71,72,94,95,96,97,98,99]
*Pleurotus ostreatus*	Oyster mushroom/Dhingri/wood fungus	Functional compound beta-glucan, nucleosides, proteins, lectins, polysaccharides, lipopolysaccharides peptides, glycoproteins, triterpenoids, lipids, phenolics (homogentisic, p-Coumaric, cinnamic acids), vitamin B, and vitamin C	Lowers cholesterol, cardiovascular disease treatment, boosts immunity, antitumor, anticarcinogenic, antimicrobial, antioxidant, hepatoprotective, antibacterial, antidiabetic, antiarthritic, and antiviral	Capsule, breadsticks, noodles	[68,71,82,83,100]
*Flammulina velutipes*	Winter mushroom/Enokitake/golden needle mushroom/velvet stem/lily mushroom/shank mushroom among different countries	Phenolic, sterols, protein, flavonoids, sesquiterpenoids (terpenoids), polysaccharides, glucan complex, lectins, peroxidases, proteases, and isoflavones	Anti-inflammatory, antibacterial, lowering cholesterol, immunomodulating, antiviral, antitumor, antioxidant, antihypertensive, and antifungal	Capsules, meat products (goat meat nuggets), mutton nuggets	[82,83,101,102]
*Grifola frondosa*	Maitake in Japanese/hen of the woods/hui-shu-hua in Chinese	Polysaccharides, beta-glucans, linoleic acid, triacylglycerols, ergosterol, steroids, homoglucans, heteropolysaccharide, D fraction ergothioneine, grifolan, lectins, oleic acid, and cyanhydric acid	Antitumor, antivirus, hepatoprotection, reducing blood lipid activities, immunomodulation, antioxidation, antiaging, and antihyperglycemia	-	[82,83,86,103]
*Lentinula edodes*	Shiitake	Beta-D-glucan, polysaccharide, lentin, adenine derivates agaritine, ergothioneine, eritadenine formaldehyde, galactose glucose, lectins, lentinamyicin, lentinan, mannose, polyisoprenoid alcohols RNA from spores statins, phenolics (p-hydroxybenzoic, gallic, 3,4 dihydroxybenzoicacids), vitamins (vitamin B_2_, vitamin B_9_, vitamin B_1_, vitamin B_3_, and vitamin B_5_), and minerals (silver (Ag), aluminum (Al), cadmium (Cd), chromium (Cr), cesium (Cs), copper (Cu), iron (Fe), mercury (Hg), potassium (K), magnesium ((Mg), molybdenum (Mo), lead (Pb), selenium (Se), zinc (Zn), and calcium (Ca))	Antibiotic, antiviral, potential agent in metabolic syndrome, hyperlipidemia treatment, depressed immune function (including AIDS), anticarcinogenic, anticancer, antiallergic, antifungal, antiviral, bronchial inflammation, heart disease treatment, controlling hypertension, antidiabetes, hepatitis treatment, and regulating urinary inconsistencies	Capsules, muffins	[69,71,82,86,100,103]
*Hericium erinaceus*	Lion’s mane mushroom/Yamabushitake	Glycans, glycoproteins, ergosterol, polysaccharides, hericenones, erinacines, proteins and peptides, beta-glucan, hericenones I, J and 3-hydroxyhericenone F, hericenone L and K, erinacerins A, B, M, and N, erinarol, cyathane-type diterpenoids, octadecenoic acid, vitamins (carotenoids, vitamin C, vitamin E, and vitamin D), lectins, monochlorobenzenes compounds, 4-pyranones, and statins	Antioxidative, anti-inflammatory, anticancer, antimicrobial, antihyperglycemic properties, treatment of neurodegenerative diseases (Parkinson’s disease), blood lipid-lowering, immunostimulant, hypoglycemic activity, antidiabetic, and antifatigue	Noodles, fermented juice	[40,58,60,85,103,104,105,106]

## Data Availability

No new data were created or analyzed in this study. Data sharing is not applicable to this article.
